# Reinforcing the physical properties of PVA/PVP with ferrous chloride for optoelectronic and antibacterial applications

**DOI:** 10.1038/s41598-025-25772-0

**Published:** 2025-11-27

**Authors:** Sh. S. El-Khiyami, A. M. Ismail, R. S. Hafez

**Affiliations:** 1 Physics Department, Faculty of Science, Cairo University, Cairo, 12613 Egypt; 2 Spectroscopy Department, National Research Centre, 33 El Bohouth Street, Dokki, 12622 Giza Egypt

**Keywords:** PVA/PVP/FeCl_2_, Optical properties, Thermal properties, Electrical properties, Antibacterial activity, Chemistry, Materials science

## Abstract

The present study investigated the composition, physical characteristics, and antibacterial efficacy of Polyvinyl alcohol/Polyvinylpyrrolidone (PVA/PVP) composite films containing different percentages (0–12%) of ferrous chloride (FeCl₂) by the solution casting method. XRD and FT-IR measurements indicate a significant increase in amorphous and substantial interactions between the polymer and filler with the addition of FeCl_2_. The composites exhibited enhanced thermal stability as evidenced by TGA, with the residual weight at 800 °C increasing from 0.21% for the pure sample to 6.28% for the sample containing 12 wt% FeCl_2_. The optical analysis reveals a significant decrease in the direct band gap, diminishing from 5.09 eV for the pure sample to 2.76 eV at 12% FeCl_2_, hence improving its applicability in optoelectronic applications. The films exhibit paramagnetic characteristics, with a saturation magnetization (Ms) of 0.055 emu/g at a 12 wt% FeCl_2_ concentration. Hopping conduction and dielectric analyses reveal a marked improvement in both the dielectric constant and AC conductivity, attaining their highest values at a FeCl₂ concentration of 10 wt%. The barrier height decreases as the temperature and FeCl₂ content increase. Impedance spectroscopy exhibited an angled spike at low frequencies and a semicircular arc at high frequencies, indicating non-Debye relaxation behavior consistent with equivalent circuit models. Furthermore, the composites showed potent antimicrobial efficacy against both Gram-negative bacteria (Klebsiella pneumoniae and Escherichia coli) and Gram-positive strains (Staphylococcus aureus and Bacillus subtilis), suggesting their potential for multifunctional optoelectronic and antimicrobial applications.

## Introduction

In order to obtain the required mechanical, chemical, thermal, or physical properties, two or more distinct polymers are physically blended without creating chemical linkages^1^. By doping polymers, we can create new and improved materials with enhanced properties^2–6^. Amorphous-crystal interfacial processes provide polyvinyl alcohol, a semicrystalline polymer, with a unique physical characteristic. PVA is a biodegradable, nontoxic polymer^7–9^ that improves electrochemical properties because hydroxyl groups bond between carbon chain backbone molecules.

A water-soluble conjugated polymer (PVP) possesses several desirable qualities, including high environmental stability, complex-forming capabilities, ease of processing, and thermal conductivity^10–13^. The proposal suggested combining PVP with PVA to decrease crystallinity and enhance permeability. A synthetic PVA/PVP polymer blend is critical for various applications, including biological medical applications^14,15^, electrochemical devices^11^, composite membranes^15,16^, tissue engineering^17–19^, wound healing^18–21^, and heat transfer^22^. The blend of PVA and PVP in a specific composition (1:1) demonstrated the most substantial charge storage capability, the highest conductivity value, and acceptable dopant addition sensitivity^15,23,24^.

Furthermore, several attempts have been made to create composite materials using PVA and PVP doped with additional fillers^25–27^. Fillers enhance the polymeric material’s electrical, thermal, optical, mechanical, and structural properties, which reduces costs and provides total control over the material’s characteristics. Furthermore, the incorporation of metal salt-containing fillers within the PVA/PVP blended matrix will assist in the development of advanced energy storage applications and promising electro-optical devices, including metal batteries, fuel cells, and capacitors, which are easy to manufacture and can be tailored to the particular requirements of the application^28–32^.

Metal halides, such as FeCl₂, CuCl₂, and NiCl₂, are used to enhance the electrical properties of polymers through ionic polarization and hopping conduction. Among them, FeCl₂ stands out due to its paramagnetic nature, variable oxidation states, and ability to form coordination bonds with polymer functional groups. Despite its potential, systematic studies on FeCl₂-doped PVA/PVP blends remain scarce. Unlike Fe₃O₄ or Fe₂O₃ nanoparticles, which often aggregate and reduce transparency, FeCl₂ can disperse uniformly within the polymer matrix, allowing for the controlled modulation of electrical and magnetic properties. There is still limited systematic research on the addition of FeCl₂ to PVA/PVP blends, especially those that link its effects on structural, thermal, dielectric, optical, magnetic, and antibacterial characteristics in a unified framework.

In previous work, El-Mahalawy et al.^33^ found that adding Ni(OAc)_2_ to the PVA/PVP blend increased electrical conductivity from 1.83 × 10^−9^ to 7.52 × 10^−7^ Ω^−1^ cm^−1^ and decreased the energy gap from 4.623 to 4.294 eV. Sadiq et al.^34^ investigated a polymer nanocomposite composed of PVP-PVA- NaCHO_3_/r-GO and found that when r-GO content is added, it has been observed that the BPNC films’ dc electrical conductivity increases, achieving a highest value of approximately 10^–6^ S/cm at 15 wt% r-GO content. Mohammed et al.^35^ investigated PVA/PVP doped with MgO, where doping reduces the band gap from 5.26 eV to 4.96 eV. Ali et al.^36^ investigated the optical and dielectric properties of PVA/PVP/LiMn_2_O_4_ and found that the band gap shrank from 5.24 eV to 4.86 eV. Zidan et al.^29^ showed that doping PVA/PVP with MWCNTs reduced the optical band gap from 5.06 eV to 4.46 eV. AlAbdulaal et al.^37^ investigated the optical performance of PVA/PVP/Gd_2_O_3_ composite films for application in electrical and laser cutoff filters. The band gap drops from 4.60 eV to 4.52 eV. Al-Ramadhan et al.^38^ found that the band gap dropped from 4.80 eV to 3.92eV when Ag nanoparticles were introduced to a PVA/PVP blend. The antibacterial activity of PVA/PVP/CuO/vitamin B1 against Escherichia coli and Staphylococcus aureus was examined by Mallakpour et al.^39^. They discovered that the inhibition zones for S. aureus and E. coli were 13.1 and 10.9 mm, respectively.

Unlike previous research, this study investigates FeCl₂ as a potential dopant for PVA/PVP blends and carefully considers how it affects the blends’ structural, optical, electrical, magnetic, and antibacterial properties. According to this study, adding FeCl₂ to PVA/PVP significantly reduces the band gap, which decreases from 4.80 eV (undoped) to as low as 2.31 eV at the greatest FeCl₂ concentration. The addition of FeCl₂ significantly increases the dielectric constant and electrical conductivity of the composites. The new system introduced here not only showcases advancements in optical and electrical properties but also exhibits paramagnetic behavior at elevated FeCl₂ concentrations. This observation has not been documented in earlier research involving different fillers. With FeCl₂-loaded PVA/PVP films showing notable inhibiting zones against both Gram-positive and Gram-negative bacteria, the antibacterial efficacy is noticeably enhanced, but the pure blend shows no such impact. Furthermore, there is an enhancement in thermal stability, and the impedance analysis indicates non-Debye relaxation consistent with equivalent circuit models. The findings suggest that these results compare those of previous filler systems in terms of multifunctionality and application potential, positioning FeCl₂-doped PVAPVP as a notably promising candidate material for optoelectronic and antimicrobial applications.

## Materials and methods

### Chemicals

Polyvinyl alcohol** (**PVA) with molecular weight of 115,000 g/mol from Alpha Chemika in India, FeCl_2_.4H_2_O from Qualikems in India (98% purity) with a molecular weight of 198.81 g/mol, and Polyvinylpyrrolidone **(**PVP) from SISCO Research Laboratory Pvt. Ltd. in Mumbai, India, with a molecular weight of 40,000 g/mol (purity >  = 99%). None of the chemicals required purification, and the solvent used throughout the experiment was distilled water.

### Preparation of (PVA/PVP/FeCl_2_) composite films

**Fig 1:**
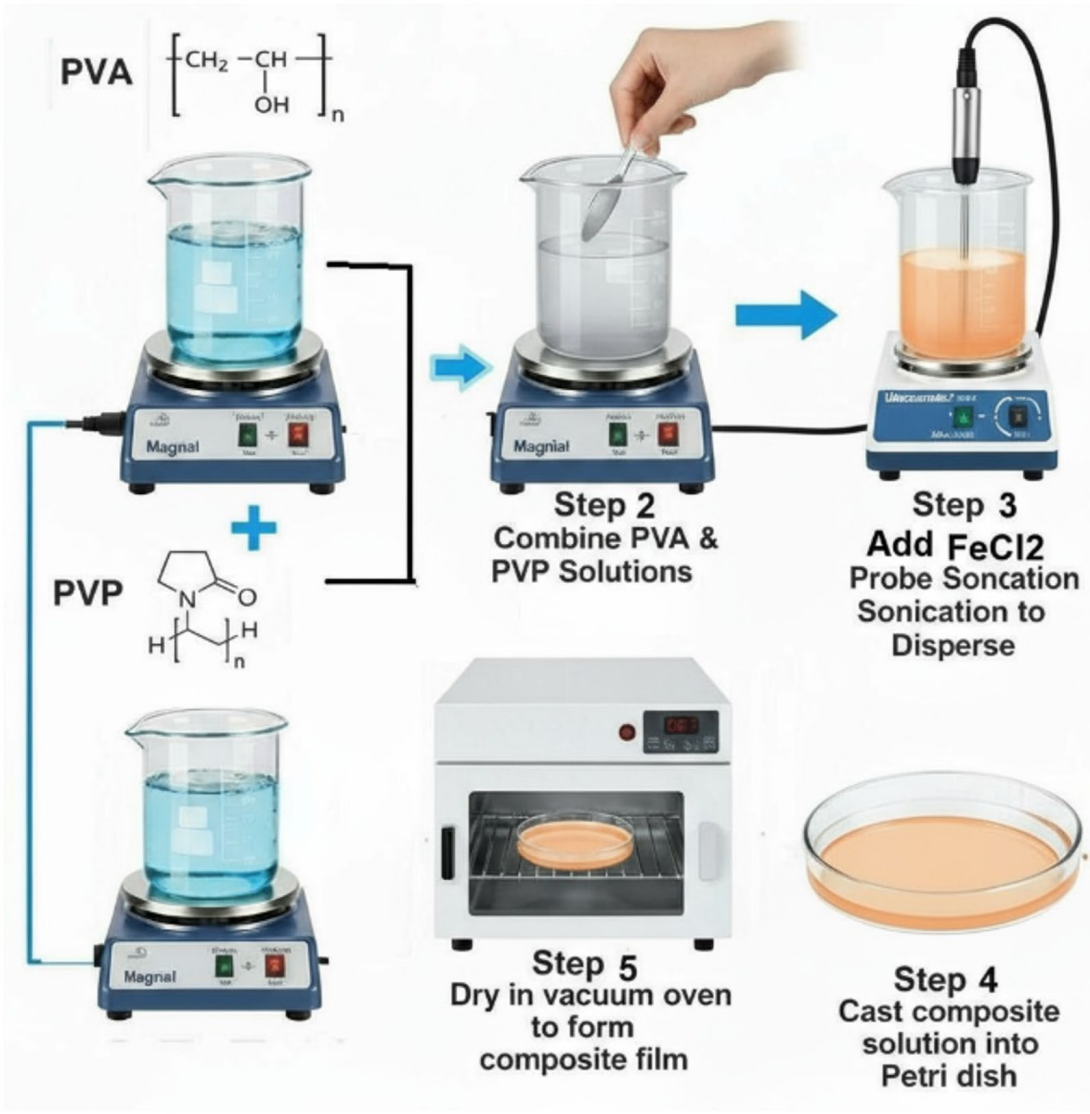
The methods for fabricating PVA/PVP/FeCl_2_ composite films using solution casting.

Composite films, which consist of (PVA) and (PVP) with varying (FeCl_2_.4H_2_O) concentrations, were created using the solution casting technique. To serve as the starting materials for the synthesis of the polymer blend, PVA and PVP were first blended in a 1:1 weight ratio, dissolved separately in 20 mL of distilled water at 70 °C, and then combined. To ensure homogeneous mixing, the solution is continuously stirred using a magnetic stirrer. FeCl_2_ was progressively added to the polymer solution at different weight percentages (4, 6, 8, 10, and 12 wt%) while keeping the ultrasonication running in order to accomplish through dispersion, decrease accumulation, ensure homogeneous doping, and create a strong bond between the metal salt and polymer chains. In order to allow the solvent to evaporate and make transparent, homogeneous films gradually, the composite solutions were carefully transferred into sterilized Petri dishes and dried in a vacuum oven set at 40 °C. The drying process was meticulously controlled to prevent issues such as cracking or bubbling. Dry films were placed in desiccators to undergo additional characterization. Figure 1 displays the Fabrication steps of PVA/PVP/FeCl_2_ composite films with a thickness of approximately 0.45 mm.

### Measurements

The X-ray diffraction (XRD) patterns were collected using an X’PERT-PRO-PANalytical channel control with a Cu-Kα target (λ = 1.5406 Å). Scans were recorded between 5° and 60° in a 2θ range. A Bruker Vertex 80 spectrometer (Germany), operating in the 4000–400 cm^-1^ spectral range, was used for Fourier transform infrared (FT-IR) measurements. A Shimadzu TGA-45H was used for thermogravimetric analysis (TGA), where the temperature was increased from ambient to 800 °C at a rate of 10 °C/min under atmospheric nitrogen pressure. UV–Vis absorption spectra were recorded in the 200–800 nm wavelength range with a Shimadzu UV-630 UV–VIS-NIR spectrophotometer. The magnetic properties of the films at room temperature were examined using a vibrating sample magnetometer (VSM) manufactured by Lake Shore (model 7410) in the United States. Dielectric measurements were performed using Novo Control Concept 40’s Broadband Dielectric Spectroscopy (BDS). Using 15 mg/ml of each composition, we assessed the antibacterial activity against gram-positive bacteria (Bacillus subtilis = B. subtilis and Staphylococcus aureus = S. aureus) and gram-negative bacteria (Klebsiella pneumoniae = K. pneumoniae and Escherichia coli = E. coli) under the same conditions. We positioned the samples on the agar medium and incubated them with light for 24 h at 37 °C. The inhibiting zone was then assessed.

## Results and discussion

### XRD analysis

**Fig. 2:**
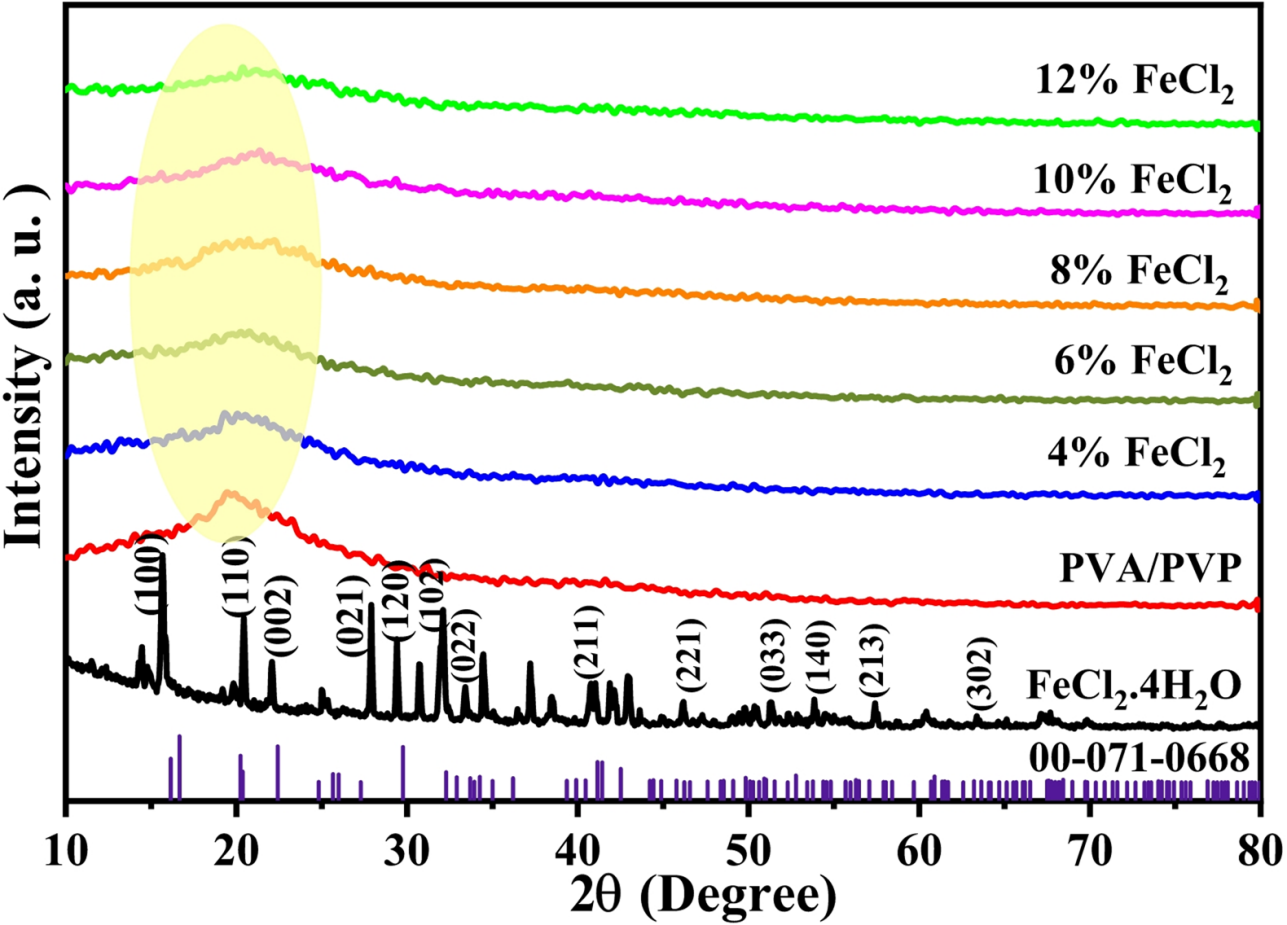
XRD pattern of hydrated FeCl_2_, PVA/PVP, and PVA/PVP doped with various quantities of hydrated FeCl_2_.

The XRD pattern was utilized to examine the internal structure of the PVA/PVP/FeCl_2_ composite films. XRD pattern of hydrated FeCl_2_ is displayed in Fig. 2, and the diffraction pattern of the hydrated FeCl2 planes is consistent with earlier research^40–42^. The PVA/PVP pattern exhibits a small, broadened hump observed at 2θ = 20° with lower intensity and sharper peaks compared to those of pure PVA^43^. The diffraction peak at 2θ = 40°, which appears in PVA, disappears, indicating a rise in the amorphous nature of PVA due to blending with PVP^33^, and a decline in peak intensity for composite films with an increase in FeCl_2_ concentration. Ions are displaced from their lattice positions by interactions between dopant ions and the functional groups of PVP and PVA. The diffracted X-ray hump broadens as a result of the displacement-induced dislocations. This implies that the host polymer becomes more amorphous as the concentration of FeCl_2_ increases^44^. A decreased energy barrier results from the reduction of the host polymer’s crystalline nature. This makes it easier for segments of the polymer to move, thereby increasing the level of ionic conductivity^45,46^. The XRD spectra show no peaks corresponding to FeCl_2_, indicating that ferrous chloride is thoroughly mixed with the polymer blend^41,47^. This enhances the complexation between the PVA/PVP blend and the filler. Figure 2 shows the indexed (hkl) powder diffraction pattern of hydrated FeCl_2_ planes of the Monoclinic, P2_1_/c, Z = 2 (PDF 01–071-0668)^40–42^. The crystallite size of the synthesized ocomposites was calculated using Scherer’s formula^48^

1 $$\text{D }=\frac{0.9 \lambda }{\beta cos\theta }$$

where *D* denotes the average crystallite size, *λ* (0.154 nm) represents the X-ray wavelength, *θ* is the Bragg diffraction angle (in degrees) corresponding to the peak maximum, and *β* refers to the full width at half maximum (FWHM) of the diffraction peak (in radians). The crystallite size of the composites was found to be 1.239, 1.342, 1.151, 1.151, 1.239, and 1.465 nm for PVA/PVP at 4, 6, 8, 10, and 12 wt%, respectively. The highest crystallite size was observed for 12% FeCl_2_. The diffraction peak at 2θ shifts slightly toward a higher diffraction angle, and when the FeCl_2_ ratio increases, peak intensity decreases, and peak broadening significantly increases. This indicates that new bonds formed between the combination and FeCl_2_ within the polymer matrix as additional bonds were broken.

### FT-IR spectroscopy

**Fig. 3:**
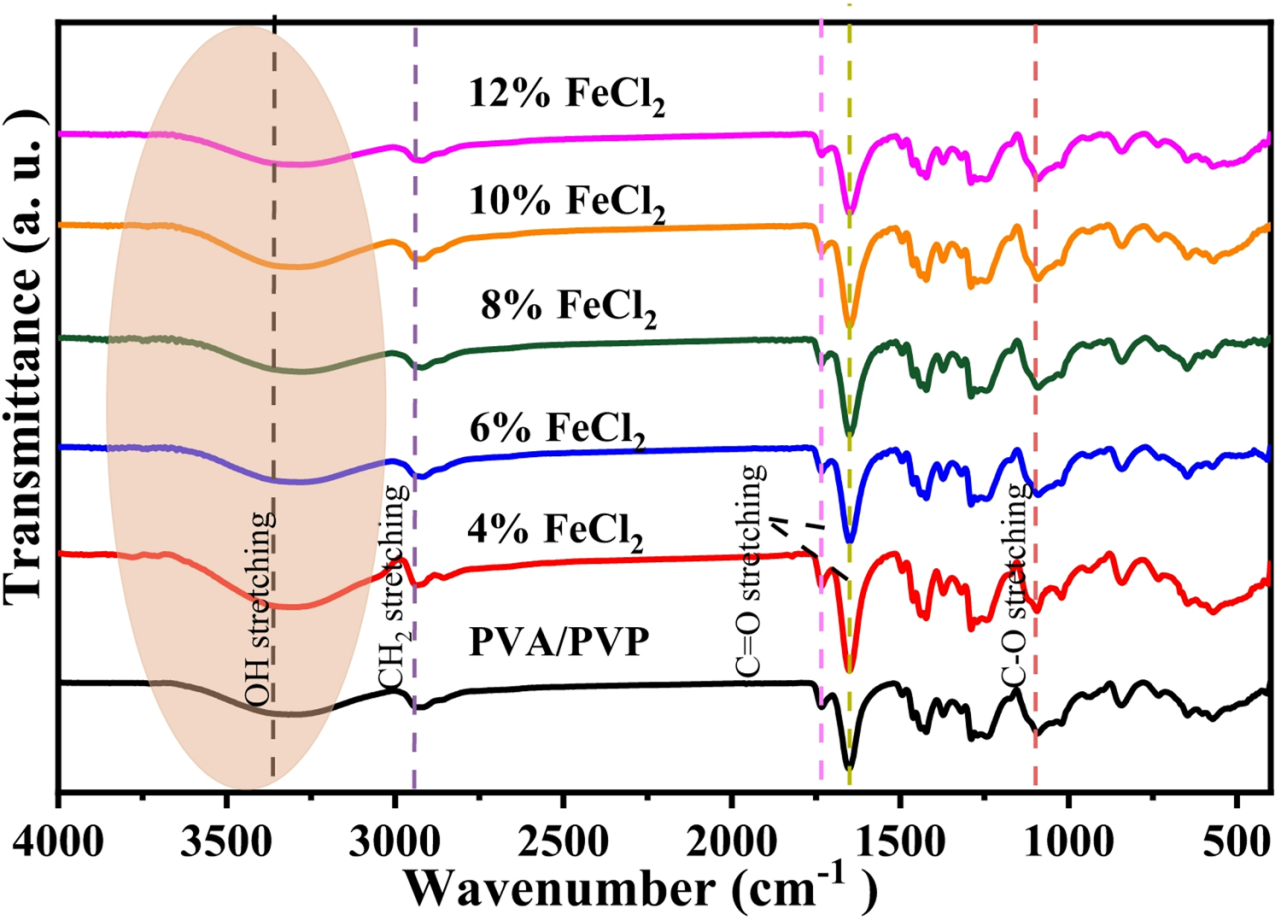
FTIR spectra of hydrated FeCl_2_, PVA/PVP, and PVA/PVP doped with various quantities of hydrated FeCl_2_.

Bond structures in polymer composites are examined using FT-IR spectroscopy. It offers details about complex development and component interactions in polymer samples. Figure 3 shows the infrared spectral profile of PVA/PVP and PVA/PVP/FeCl_2_ composite samples. The spectra agree with previously published data and contain all the essential bands found in the PVA and PVP spectra^24,33^. The band at 3346 cm^-1^ is caused by the OH group’s stretching vibration. Stretching and bending vibrations of the CH_2_ group are seen at 2917 cm^-1^ and 1421 cm^-1^, respectively. The bands that emerge at 1733 cm^-1^ and 1656 cm^-1^, respectively, demonstrate the C=O stretching of the ketone and lactam groups. The C-O stretching of the ether bond and the C-N stretching of PVP are found in the range of 1241 cm^-1^ to 1285 cm^-1^. Primary alcohol exhibits an O–H out-of-plane bending vibration at 843 cm^-1^ and a C-O stretching vibration at 1087 cm^-1^. For PVA/PVP films doped with FeCl_2_, the OH groups are hydrogen-bound, as indicated by the broadness of this band. The spectral analysis of PVA/PVP films doped with FeCl_2_ reveals significant changes in band position and intensity compared to the pristine blend sample. These changes indicate that the metal salt and the polymer blend exhibit an essential interaction. The finding demonstrates that the addition of FeCl_2_ significantly impacts the structural characteristics of the PVA/PVP blend.

### TGA analysis

**Fig. 4:**
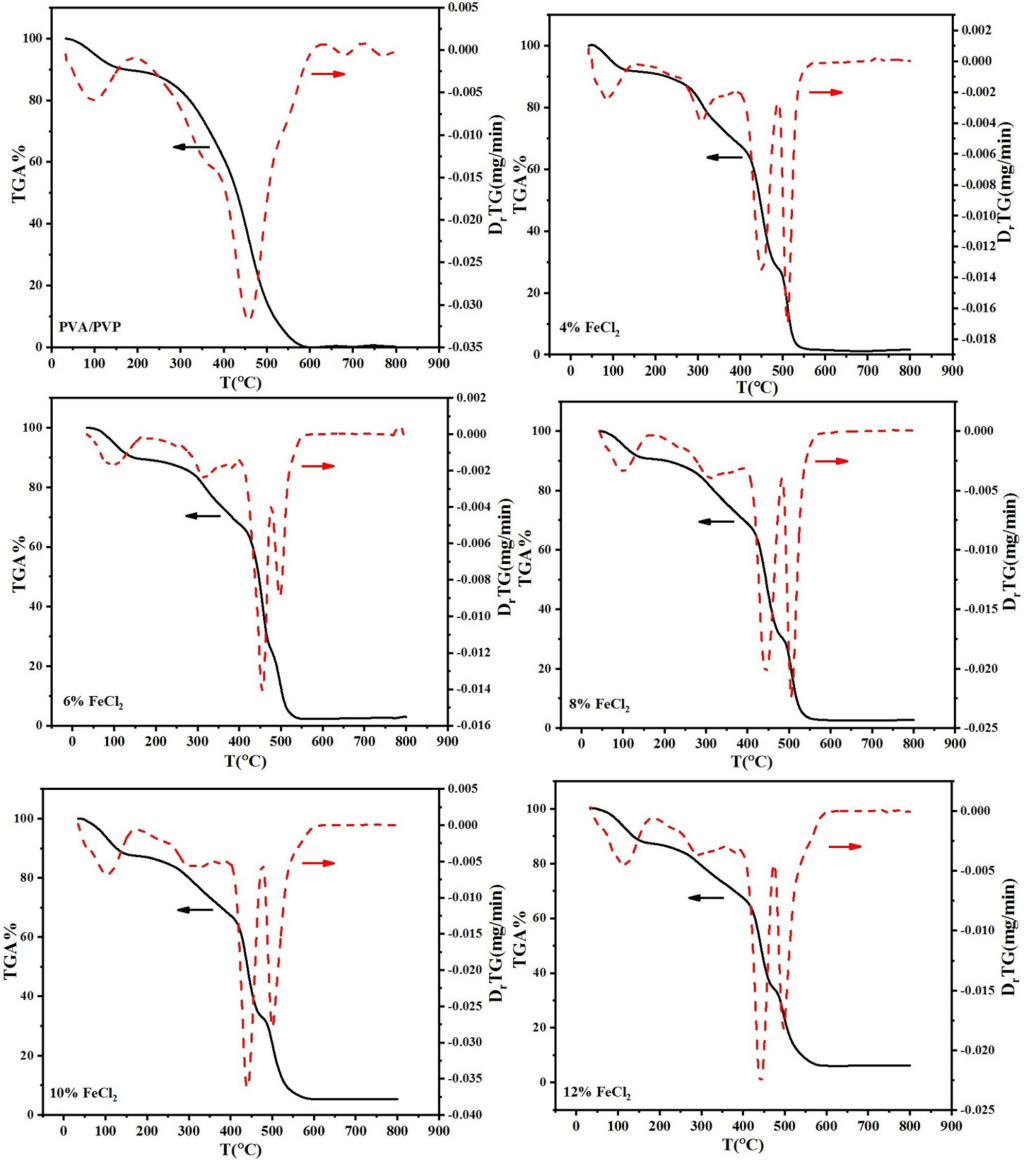
Thermogravimetric analysis and its derivative results of PVA/PVP and PVA/PVP doped with various quantities of hydrated FeCl_2_.

Thermogravimetric analysis is a powerful analytical method that accurately assesses the filler content in polymers and composites. Including fillers in these materials can markedly impact the final product’s properties, including stiffness, thermal expansion, and damping. This is particularly important for electronic applications. The Purpose of this study was to examine how FeCl_2_ affected the thermal stability of samples of PVA/PVP blends. This was accomplished by subjecting the polymer composite samples to a TGA test in a nitrogen atmosphere under controlled conditions. A broad temperature range of 25 to 800 °C was utilized for the test. Figure 4 displays the TGA and DTG curves that were produced, giving a thorough illustration of the samples’ thermal behavior. The degradation of the PVA/PVP blend film could be observed in three distinct stages. The first stage occurs between 50 °C and 190°C and is mainly characterized by an initial weight loss^49^. Removing absorbed water and solvent evaporation are the leading causes of this weight loss. The blend began to degrade as the temperature increased, with the second stage of degradation taking place between 312 and 394 °C. During this stage, it was observed that the side group (-OH) was degrading, indicating that the chemical structure of the blend was undergoing alteration^50^. Finally, the third stage of degradation, occurring between 394 and 480 °C, revealed the structural breakdown of the blend. This stage indicates the complete breakdown of the blend’s chemical structure. Table 1 presents a comparative analysis of the degradation temperatures of PVA/PVP/FeCl_2_ composite samples, along with their corresponding weight losses at 40%, 60%, and 80% weight loss. A fascinating finding resulted from the data analysis. The PVA/PVP pure blend exhibited a residual percentage of approximately 0.21% at 800 °C. A residue percentage of 6.28% was observed in the PVA/PVP blend sample doped with 12 wt% FeCl_2_. Notably, adding FeCl_2_ to the PVA/PVP blend significantly increased the samples’ thermal stability.

**Table 1:** Degradation temperatures at 40, 60, and 80% weight loss of the PVA/PVP blend-based composite and residual weight percentage at 800 °C.

FeCl_2_ Concentrations		T_40%_^o^C	T_60%_^o^C	T_80%_^o^C	Residue (%) at 800 °C
0		404.3	449.5	486.1	0.21
4		429.7	452.6	487.7	1.64
6		430.2	455.5	503.7	2.73
8		435.1	468	509.6	2.93
10		439.2	473.3	515.2	5.39
12		450.5	485.9	523.1	6.28

### Optical properties

#### UV/Vis absorption spectroscopy

**Fig. 5:**
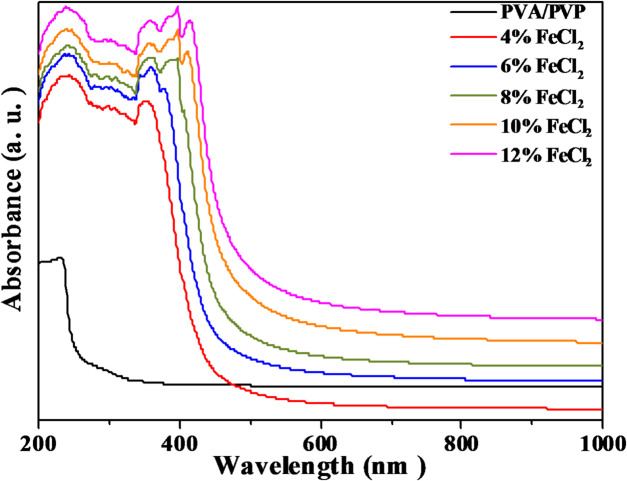
UV–Vis spectra of PVA/PVP blend filled with different concentrations of hydrated FeCl_2_.

A flexible and effective method for examining the optical characteristics of organic materials is UV/Vis spectroscopy. This non-destructive method enables us to study the interaction between light and matter, providing valuable information on the electrical structure of materials. Figure 5 shows the spectra of the PVA/PVP and PVA/PVP/FeCl_2_ composites utilized in this investigation. Analyzing the optical absorption spectra is a highly successful approach to understanding the electronic characteristics and bandgap structure of polymeric films. Because both PVA and PVP are transparent, the PVA/PVP pure blend’s absorption spectra show minimal absorption over the entire visible spectrum (400–800 nm). However, there is a slight rise in absorbance when the wavelength decreases from 400 nm to approximately 228 nm. An absorption peak has been detected in the 215–238 nm wavelength range. This is caused by segments containing a carbonyl group and assigned to n-π* transitions^51^.

Furthermore, a shoulder-like peak at 288 nm can be found in the absorbance spectra of the PVA/PVP pure blend. This peak is linked to the π-π* transition in unsaturated bonds, such as C = C and/or C = O in the polymer^52^. The absorbance spectra of PVA/PVP/FeCl_2_ composite samples exhibit extra peaks at 361, 383, and 418 nm, which can be ascribed to the existence of octahedral and tetrahedral structures of ferrous chloride^53^. These additional bands indicate that the composites have distinct structural characteristics, which could affect their performance in different applications. Additionally, Fig. 5 demonstrates an apparent increase in film absorbance with increasing metal salt concentration. This observation confirms the considerable interaction between PVA/PVP and FeCl_2_ metal salt particles.

#### Optical energy gap

**Fig. 6:**
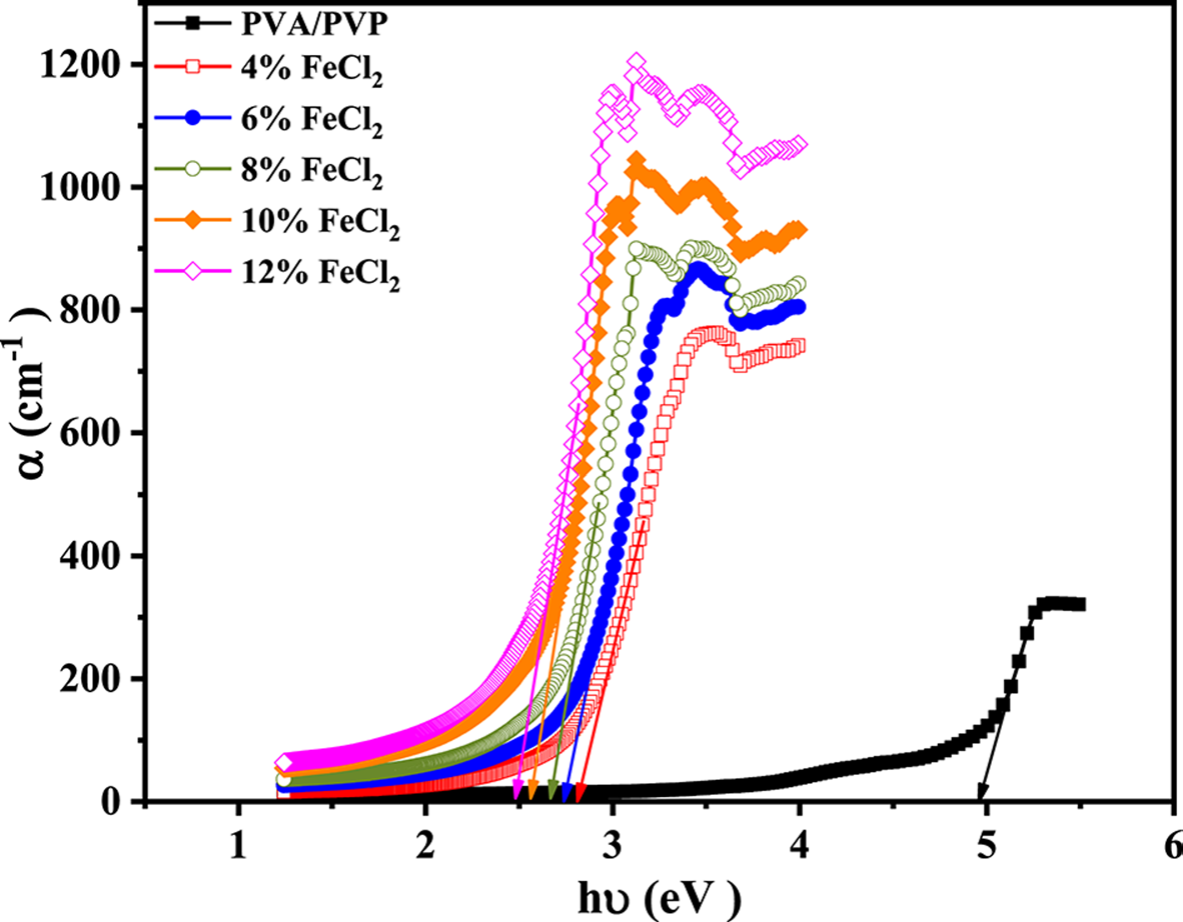
Absorbance coefficient (α) versus photon energy (hυ) for PVA/PVP blend filled with different concentrations of hydrated FeCl_2_.

An important measure that is dependent on the light’s wavelength is the absorption coefficient (α). It provides a precise knowledge of the decrease in incident light intensity as it moves through the material being studied. A formula, Beier-Lambert’s law, calculates the absorption coefficient^54^.

2 $$\alpha \left( \lambda \right) = { }2.303\frac{A}{t}$$

where A is the optical absorbance and t is the thickness of the sample. Figure 6 provides the graphical representation of the α value for all studied samples. The calculated and tabulated absorption edge values for the composite films are shown in Table 2. According to the experimental findings, when the FeCl_2_ content increases from 0 to 12%, the absorption edge value of the composite films drops from 4.80 eV to 2.45 eV. Ferrous chloride’s presence in the PVA/PVP blend is ​​the leading cause of this decrease in the absorption edge value. The observed red shift in the absorption edge, when combined with XRD data, results in a narrowing of the bandgap transition and a decrease in the average crystallite size.

**Table 2:** The values of the absorption edge, indirect (E_g(indirect)_), and direct (E_g(direct)_) band gap of the PVA/PVP blend-based composite.

FeCl_2_ Concentrations	Absorption edge (eV)	E_g(indirect)_ (eV)	E_g(direct)_ (eV)
0	4.80	4.60	5.09
4	2.80	2.60	3.07
6	2.70	2.50	3.00
8	2.60	2.45	2.82
10	2.50	2.35	2.71
12	2.45	2.31	2.76

Using Tauc’s model, we used the following relation to obtain the optical band gap (E_g_)^55^

3 $$\left( {\alpha {\text{h}}\nu } \right)^{{\text{m}}} = \beta ({\text{h}}\nu - {\text{E}}_{{\text{g}}} )$$

**Fig. 7:**
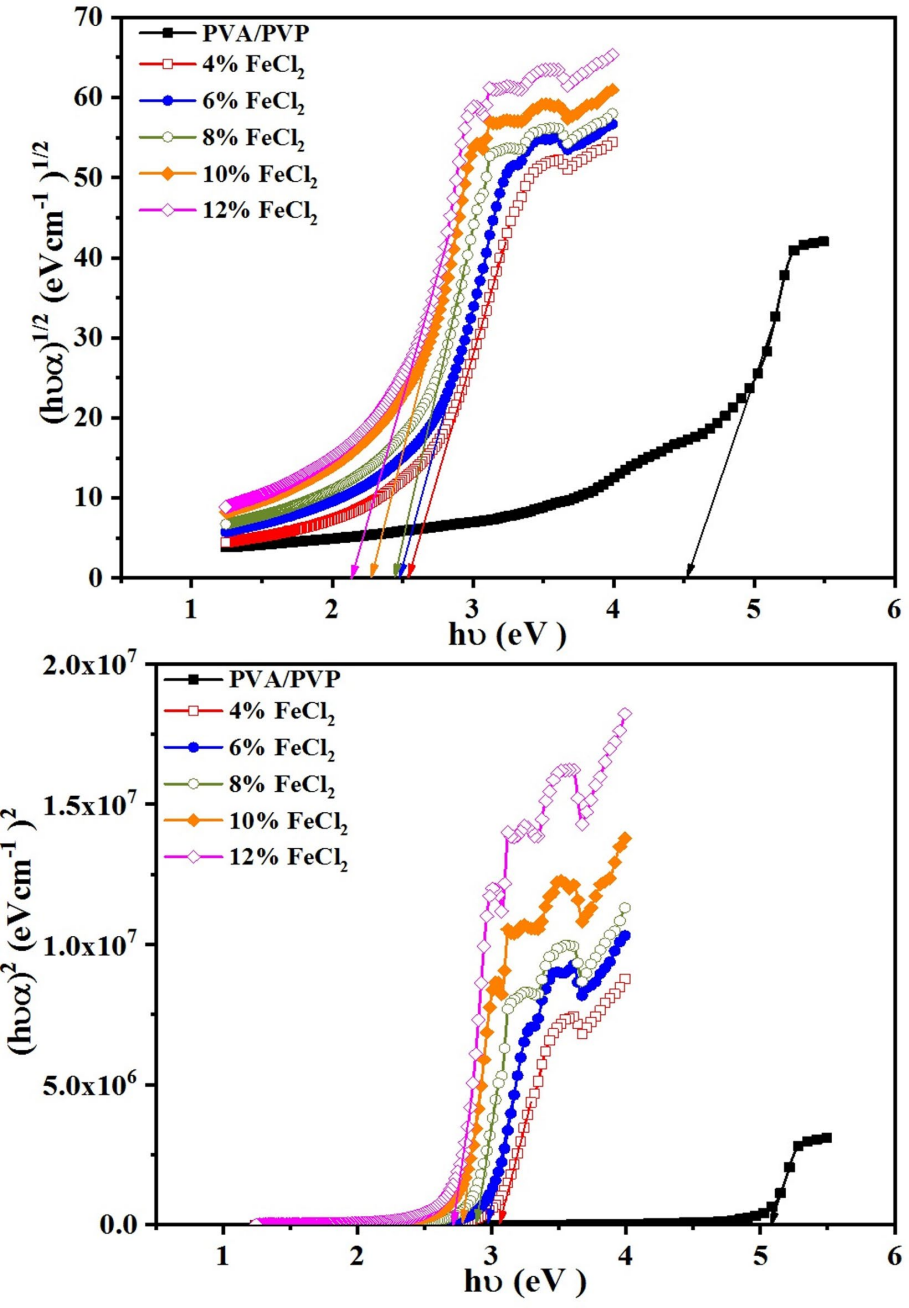
Relation between (αhυ)^1/2^ and (αhυ)^2^ versus photon energy (hυ) for PVA/PVP blend filled with different concentrations of hydrated FeCl_2_.

β is a constant, and optical transitions are characterized by m = 2 and m = 1/2, corresponding to direct and indirect bandgap behaviors, respectively. To evaluate the direct and indirect band gaps, one must plot the relationship between (αhʋ)^1/2^ and hʋ for the indirect band gap and (αhʋ)^2^ and hʋ for the direct band gap. These plots are seen in Fig. 7. Following this, zero absorption can be achieved by extrapolating the straight section of the curves. The obtained direct and indirect band gap energies generally decrease as the FeCl_2_ content rises, as Table 2 illustrates. The decrease in band gap energy is attributed to hydrogen bonding, which primarily occurs between the Fe ions and the hydroxyl groups^56^. Incorporating FeCl_2_ metal salt into the PVA/PVP matrix results in localized states and a decreased optical band gap, as evidenced by the observed overlaps. Designing novel composite materials for a range of industrial and technological applications may be impacted by these discoveries.

### Magnetic properties

**Fig. 8:**
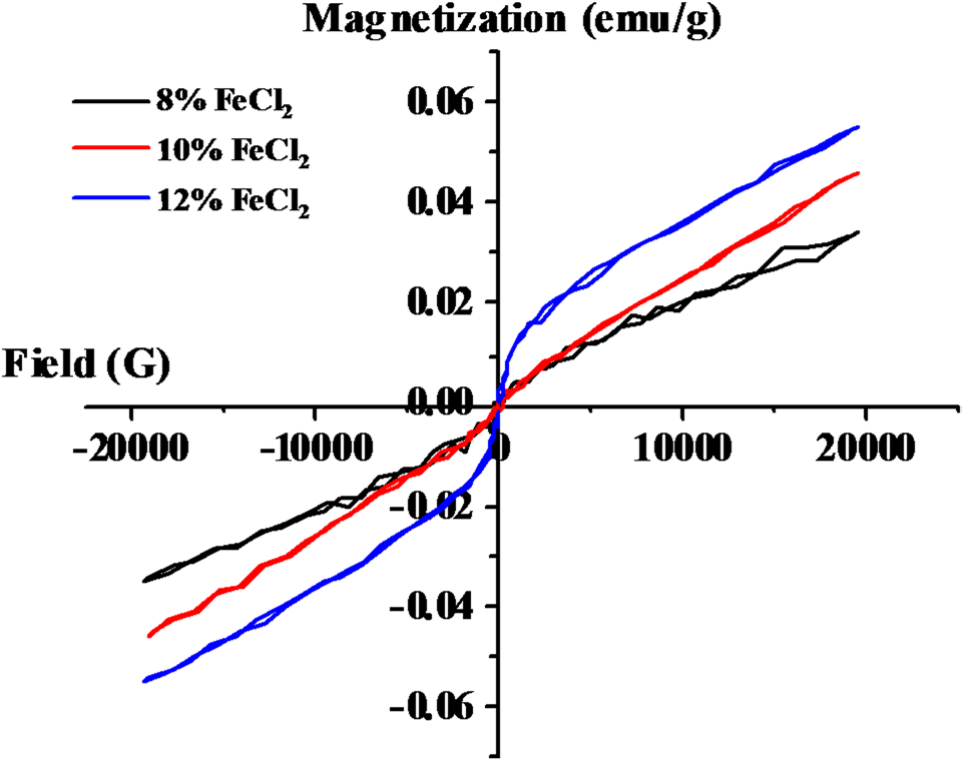
Hysteresis loops for PVA/PVP blend filled with different concentrations of hydrated FeCl_2_.

The magnetic hysteresis loops of the PVA/PVP/FeCl₂ composite samples measured at 300 K are presented in Fig. 8. All composites containing 8 wt% or higher FeCl₂ exhibit paramagnetic behavior. The magnetization curves show no noticeable hysteresis and pass through the origin, indicating the absence of remanent magnetization and coercivity. This confirms that the samples are paramagnetic in nature, consistent with the intrinsic magnetic characteristics of FeCl₂^57^. The saturation magnetization (Ms) values are found to be 0.034 emu/g for 8% FeCl₂, 0.046 emu/g for 10% FeCl₂, and 0.055 emu/g for 12% FeCl₂. The saturation magnetization increases by approximately 62% when the FeCl₂ content rises from 8 wt% to 12 wt%, indicating that the magnetic response scales proportionally with the filler concentration. The progressive increase in Ms with increasing FeCl₂ concentration is caused by an increased percentage of magnetic ions integrated into the polymer matrix. Furthermore, the PVA/PVP polymer matrix may restrict the mobility of the magnetic dipoles, leading to an increased energy barrier for magnetization. This restriction can result from strong interfacial interactions and the partial immobilization of Fe^2^⁺ ions within the polymeric network.

### Electrical properties

The dielectric properties were measured using a Novocontrol Concept 40 s Broadband Dielectric Spectrometer. This system was utilized to conduct dielectric measurements by applying an AC voltage across a material sample and measuring the resultant current, which determines both the amplitude and phase of the current and voltage. The system automatically calculates the complex impedance, the real (ε′) and imaginary (ε″) parts of the dielectric permittivity, and the AC conductivity (σac). The samples were cut to the required dimensions, and each sample was precisely positioned between two parallel electrodes to establish a parallel-plate capacitor configuration. The characteristics of the material across a range of frequencies and temperatures were determined.

### Dielectric constant

**Fig. 9:**
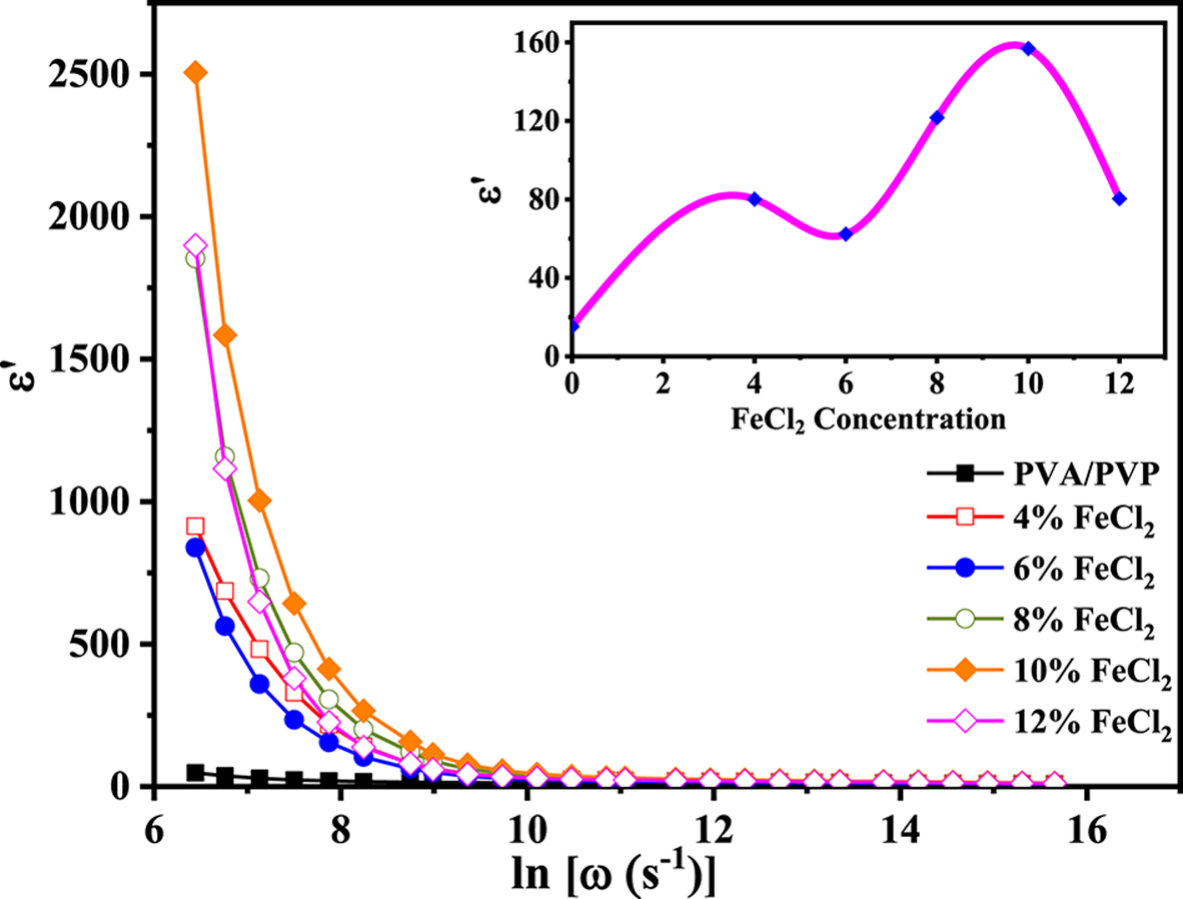
The frequency dependence of the dielectric constant for the PVA/PVP blend filled with different concentrations of hydrated FeCl_2_ at a selected temperature of 70 °C.

The dielectric constant (ε') of PVA/PVP/FeCl_2_ composite films exhibits frequency-dependent behavior, as demonstrated by the data shown in Fig. 9. As frequencies increase, the polarization of the space charge decreases, lowering the total polarization. Notably, ε' reduces with increased frequency, validating that the dielectric constant has a high starting value for polar materials. The value of ε' starts to decrease as the AC field’s frequency rises^58^. At higher frequencies, the behavior of ε' appears to be independent of frequency. The electrical relaxation mechanisms may be responsible for this. However, ε' exhibits a sudden rise in frequency, suggesting the presence of space charge polarization in the low-frequency range. The increase in ferrous chloride content results in a rise in the dielectric constant ε' of composite samples caused by forming a continuous network of metal salt ions inside the films. The insert of Fig. 9 provides a clear visual representation of this behavior. When the concentration of FeCl_2_ is around 10 wt% or higher, it hinders the flow of charge carriers in the conducting pathway by creating blockages caused by increasing filler content within the composite sample. Consequently, there is a reduction in the ε value^59^.

### Conductivity relaxation

**Fig. 10:**
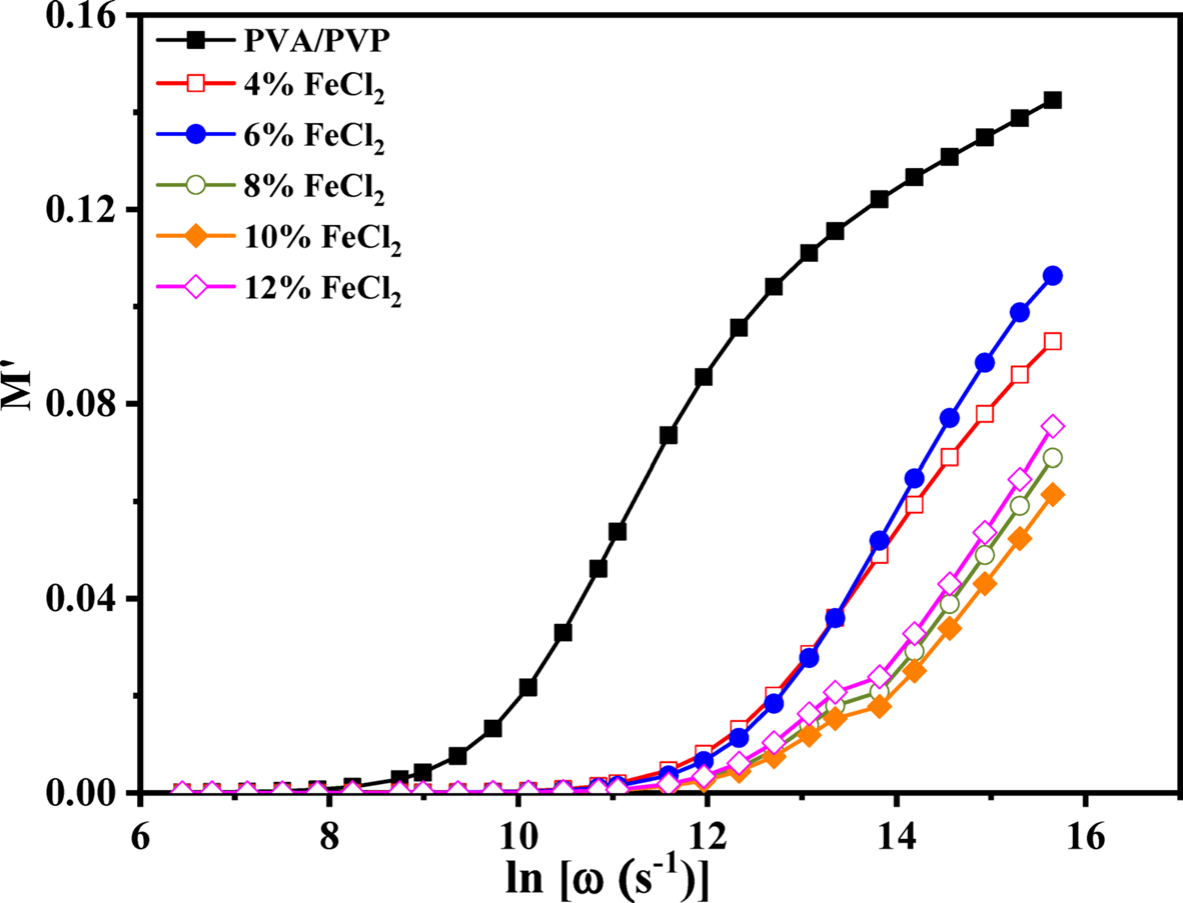
The frequency dependence of M′ for PVA/PVP blend filled with different concentrations of hydrated FeCl_2_ at a selected temperature of 70 °C.

**Fig. 11:**
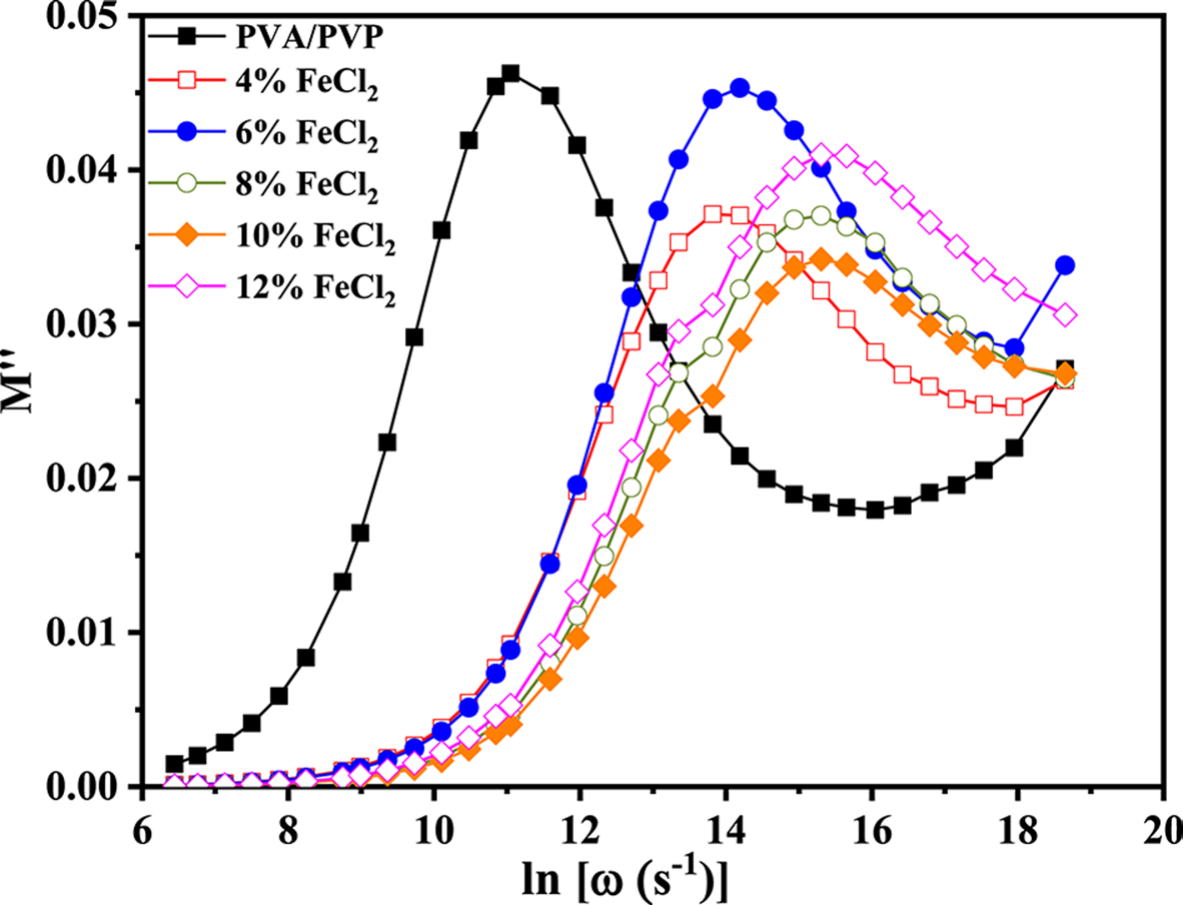
The frequency dependence of M″ for PVA/PVP blend filled with different concentrations of hydrated FeCl_2_ at a selected temperature of 70 °C.

The following formula is used to calculate the complex dielectric modulus (M*)^6,60^.

4 $${\text{M}}^{*} \left( \omega \right) \, = \frac{1}{{\varepsilon^{*} \left( \omega \right)}} = {\text{ M}}^{\prime } \left( \omega \right) \, + {\text{ i M}}^{\prime \prime } = \frac{{\varepsilon^{\prime } }}{{\varepsilon^{\prime 2 } + \varepsilon^{\prime \prime 2} }} + {\text{ i}}\frac{{\varepsilon^{\prime \prime } }}{{\varepsilon^{^{\prime}2 } + \varepsilon^{\prime \prime 2} }}$$

where M’ and M’' represent the real and imaginary components of the dielectric modulus. Figure 10 illustrates the relation between frequency and the dielectric modulus M’s actual component for PVA/PVP/FeCl_2_ composite samples. The graph depicts the conductivity of the ionic type, represented by an S-shaped curve^61^. Figure 11 illustrates the connection between frequency and the imaginary component of the dielectric modulus, or M’', for composite samples. As the temperature increases, the bell-shaped peaks shift toward higher frequencies. This is acceptable when the resistive and/or capacitive study shows an abundance of localized relaxation.

**Fig. 12:**
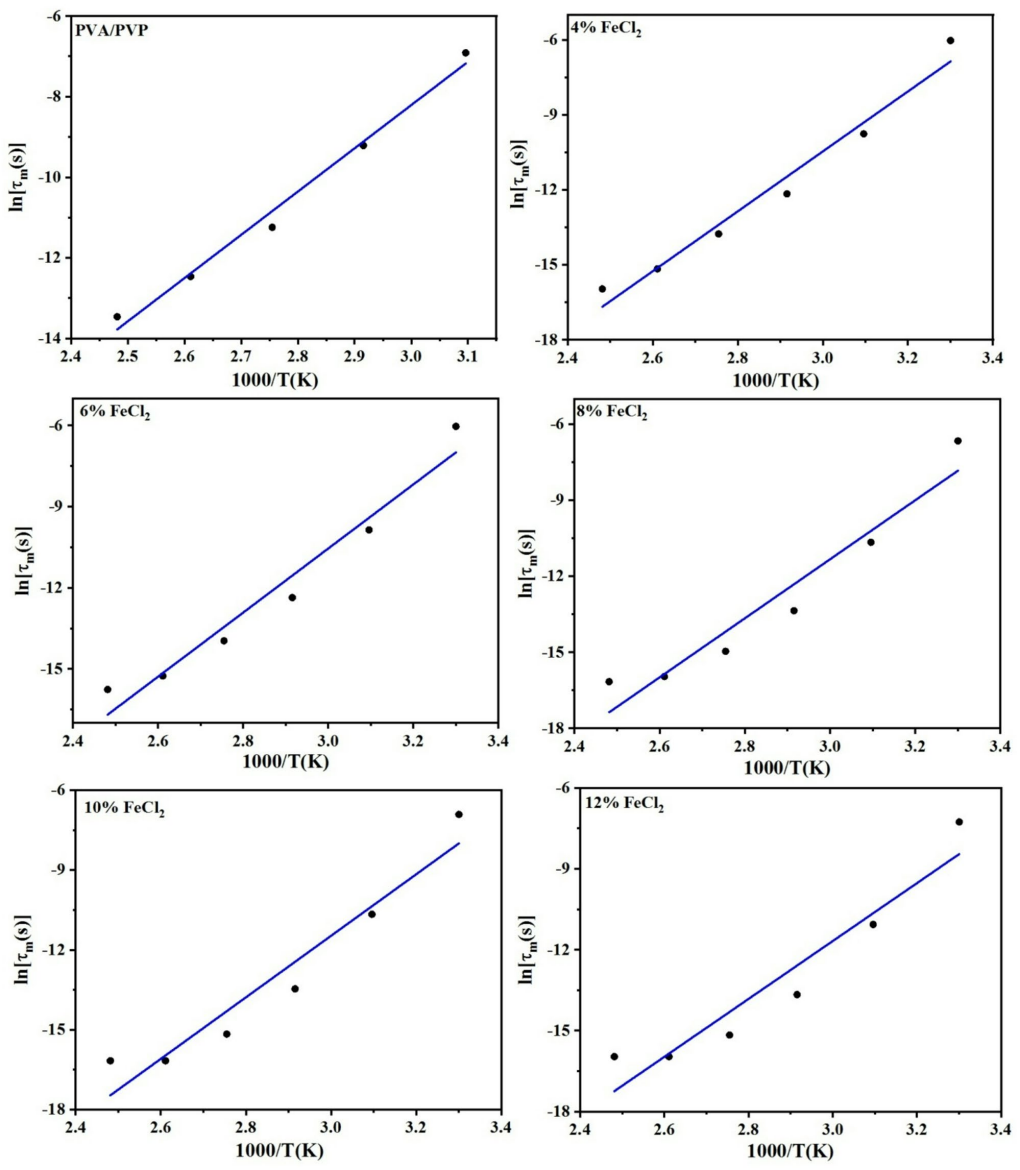
The variation of ln [τ_m_(s)] versus 1000/T (K) for a PVA/PVP blend filled with different concentrations of hydrated FeCl_2_.

The relaxation time (τ_m_) can be resolved by identifying the frequency (ω_m_) at which the maximum M’'_max_ takes place, such that ω_m_τ_m_ = 1. Figure 12 shows a plot of relaxation time versus reciprocal temperature, which was fitted using the following equation,

5 $$\tau = {\tau }_{0} {e}^{\Delta E/kT}$$

where τ_o_ is the high-temperature limit of the relaxation time, k is the Boltzmann constant, T is the absolute temperature, and ΔE is the obtained activation energy. The most likely conduction mechanism is charge carrier hopping, as shown by the activation energy along with relaxation time values for each sample in Table 3. As the FeCl_2_ ratio increased, the activation energy reduced.

**Table 3:** Activation energy ΔE (eV) and the high-temperature limit of the relaxation time τ(s) for conductivity relaxation.

FeCl_2_ Concentrations	ΔE_relax. M''_ (eV)	τ_relax. M''_ (s)
0	0.94	8.41 × 10^–18^
4	1.03	7.04 × 10^–21^
6	1.01	9.77 × 10^–21^
8	1.00	8.02X10^–21^
10	0.99	9.30X10^–21^
12	0.92	8.88X10^–20^

### AC Electrical conductivity

#### Temperature-dependent

**Fig. 13:**
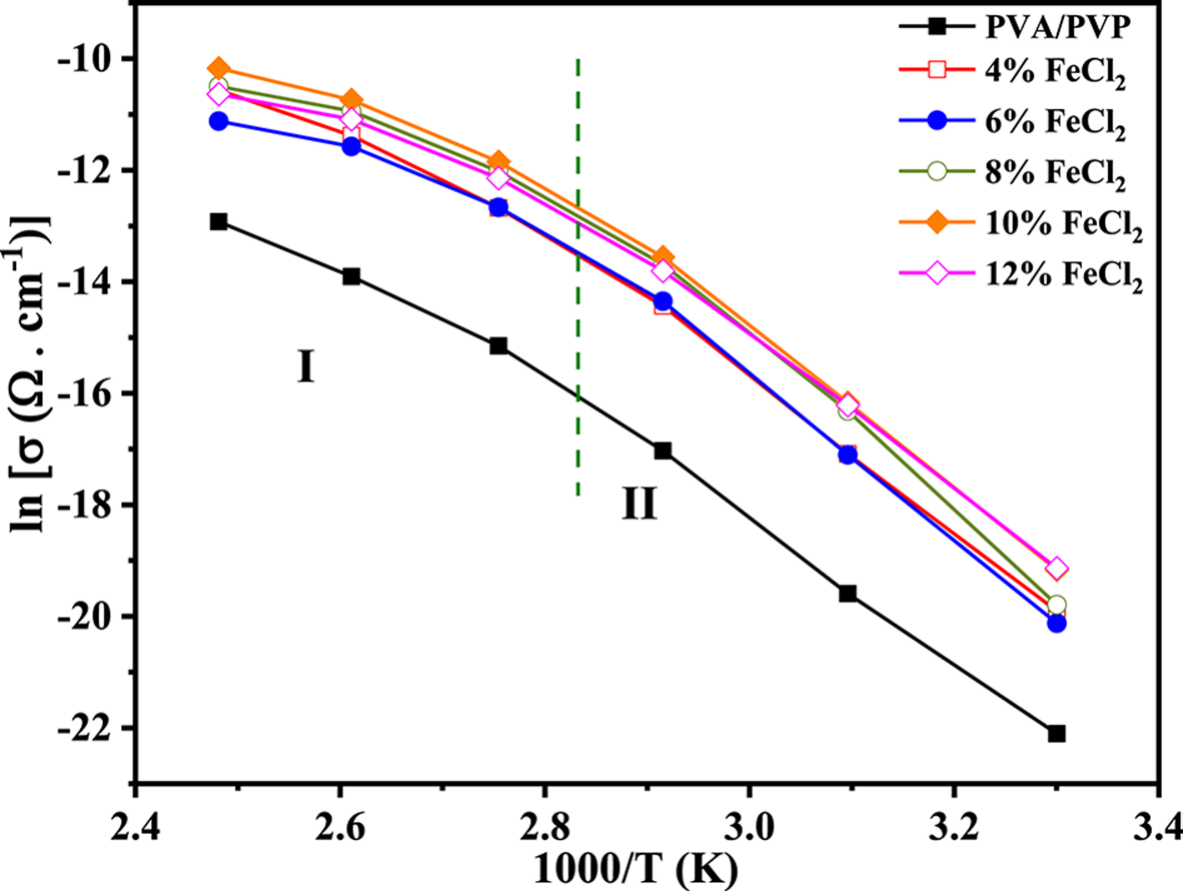
The variation of ln(σac) versus 1000/T (K) for a PVA/PVP blend filled with different concentrations of hydrated FeCl_2_ at 1 kHz.

Figure 13 shows the ac conductivity plot of PVA/PVP/FeCl_2_ films, plotted with 1000/T(K) at 1 kHz using the following Arrhenius equation.

6 $$\sigma_{{{\text{ac}}}} = {\text{ A}}_{0} {\text{exp }}\left( { - \Delta {\text{E}}/{\text{kT}}} \right)$$

where A_0_ is the pre-exponential factor, ΔE is the activation energy, and k and T are Boltzmann’s constant and the absolute temperature, respectively. Figure 13 shows that as the temperature increases, the conductivity of the investigated samples also rises. At higher temperatures, ion hopping can be related to conductivity values where more carriers participate^62,63^. In contrast to pure PVA/PVP film, the conductivity of composite samples is significantly higher. This occurrence is attributable to the increased mobility of charge carriers, as more can move freely across the amorphous areas of the PVA/PVP matrix, thereby escaping from the traps. The highest conductivity is observed at a dopant concentration of 10 wt% FeCl_2_. Conversely, the conductivity drops below that concentration but remains higher than in a pure blend. The primary cause of the reduced mobility of charge carriers, which is most likely what results in the decrease in conductivity, is the dispersion of ionized molecular aggregates.

Analysis of the calculated values of ΔE_ac_ listed in Table 4 reveals that the hopping conduction mechanism is responsible for ac conduction in all composite samples. The observed low values of activation energy support this conclusion. Notably, the transport of ions through the polymer matrix is primarily influenced by the FeCl_2_ concentration and the dielectric constant^64^. These results imply that the composite material’s ac conduction behavior can be effectively controlled by adding FeCl_2_ to the polymer matrix.

**Table 4:** The dependence of the activation energy (ΔE) for the PVA/PVP blend on FeCl_2_ concentration at 1 kHz.

FeCl_2_ Concentrations	Δ E(eV) region II (343K-303K)	Δ E(eV) region I (403 K- 363 K)
0	1.36	0.70
4	1.28	0.66
6	1.29	0.53
8	1.25	0.48
10	1.23	0.47
12	1.26	0.50

#### Frequency-dependent

**Fig. 14:**
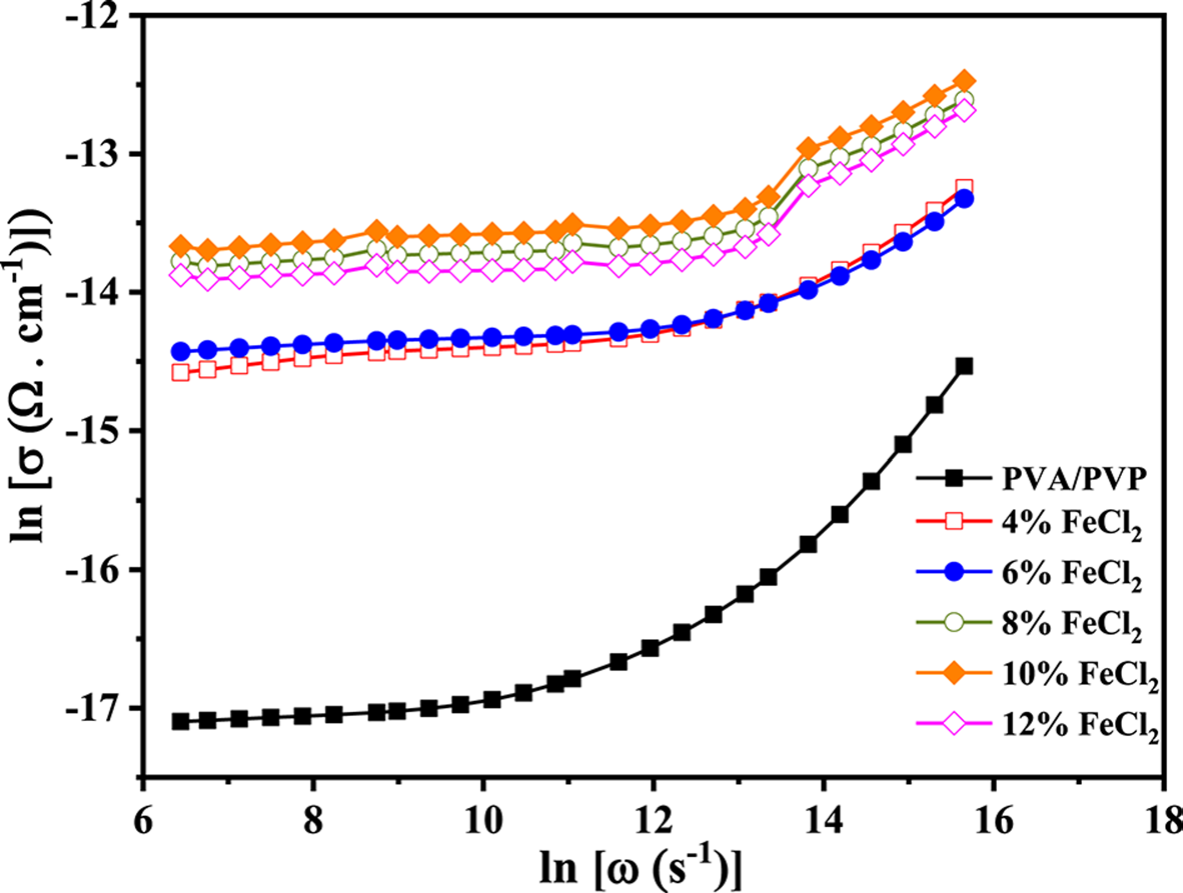
The frequency dependence of ln (σ_ac_) for PVA/PVP blend filled with different concentrations of hydrated FeCl_2_ at constant temperature 70 °C.

One Popular method for describing frequency-dependent conductivity is the universal dispersion relaxation (UDR)^65^

7 $$\sigma (\omega ) = \sigma_{{{\text{dc}}}} + {\text{A}}\omega^{{\text{s}}}$$

where σ_dc_ is the dc conductivity of the sample, A is a temperature-reliant constant, and s is the power law exponent. It is typically within the range of 0 < s < 1. The transport mechanism describes a thermally induced hopping process that takes place between two locations that are separated by an energy barrier^66,67^. The composites’ electrical conductivity (lnσ) at room temperature is plotted logarithmically against angular frequency (lnω) in Fig. 14. The electrical conductivity of the films increases with frequency due to charge carrier hopping and the low-frequency polarization of the space charge^68^. In addition, weak connections between the polymer and FeCl_2_ particles have been strengthened, resulting in a strong coupling across the grain boundary^69^.

**Fig. 15:**
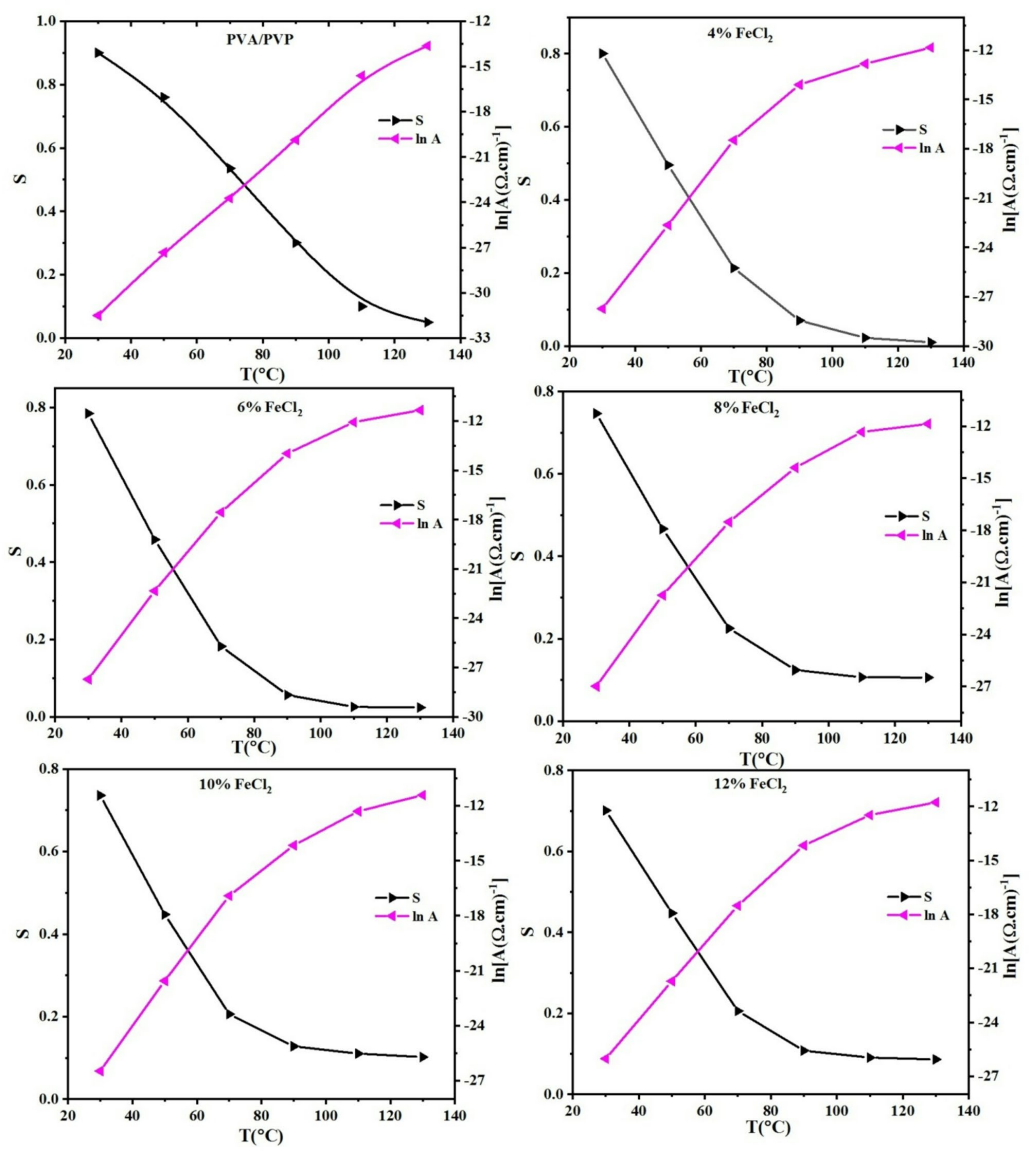
Thermal variation of the factor A and exponents for PVA/PVP blend filled with different concentrations of hydrated FeCl_2_.

Pure polyblend and its composites, Fig. 15 shows how the exponent s varies with temperature. The exponent s decreases with increasing temperature and is always in the range between 0 and 1. Based on previous research, these findings suggest that correlated barrier hopping (CBH) is the most suitable model to describe the AC conduction process in all examined films^70^. The relationship between the exponent s and temperature dependency in this model is as follows:

8 $$s=1- \frac{6kT}{{W}_{M}- {K}_{B} T Ln(\frac{1}{\omega {\tau }_{0}})}$$

The following terms define the above equation: τ_0_ represents the characteristic relaxation time of an atom’s vibrational period, which is approximately equal to 10^−13^ s. k refers to Boltzmann’s constant. The energy required to move electrons from a location to infinity is known as the maximum barrier height at infinite separation, or W_M_. This is also known as the charge carriers’ binding energy at specific positions. To approximate this correlation, the exponents turn into:

9 $$s=1- \frac{6 k T}{{W}_{m}}$$

**Fig. 16:**
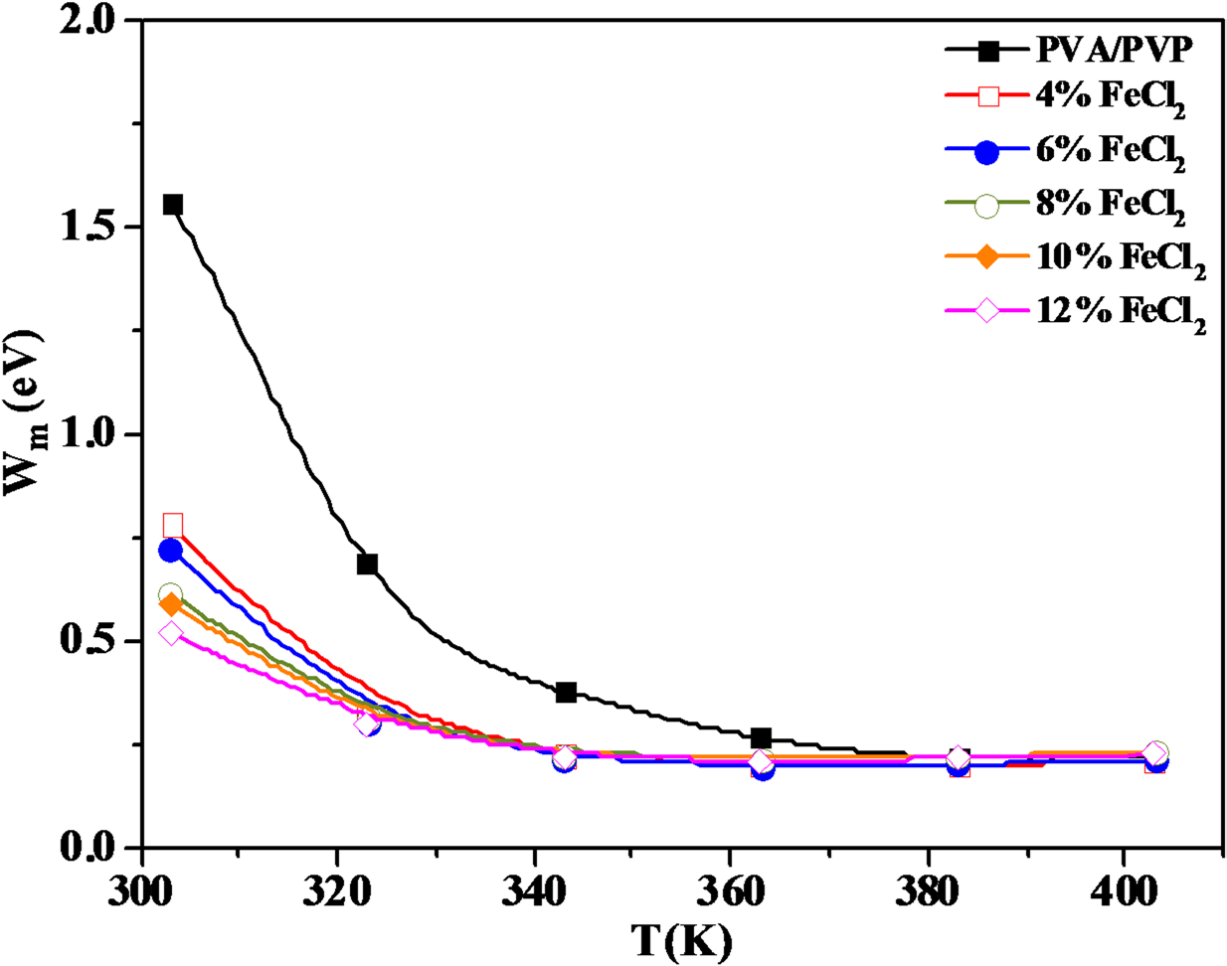
Temperature dependence of *W*_*m*_ for PVA/PVP blend filled with different concentrations of hydrated FeCl_2_.

To calculate the mean value of the binding energy, W_m_, a plot of (1-s) versus T can be utilized to calculate the slope. Figure 16 depicts the W_m_ variant as a temperature function for the PVA/PVP/FeCl_2_ composite samples. Our observations reveal that W_m_ decreases with increasing temperature and FeCl_2_ content, correlating with a decrease in the exponent s. As a consequence, more free charge carriers can cross the barrier. The increase of σac with temperature is confirmed by this behavior^71^.

In the formulation of hopping between localized states at the Fermi level, σac can be defined and assessed by^72,73^:

10 $$\sigma_{ac} = \left[ {N\left( {E_{F} } \right)} \right]^{2} T k e^{2} \alpha^{ - 5} \omega \left[ {{\text{ln}}\left( {\frac{{\nu_{ph} }}{\omega }} \right)} \right]^{4}$$

**Fig. 17:**
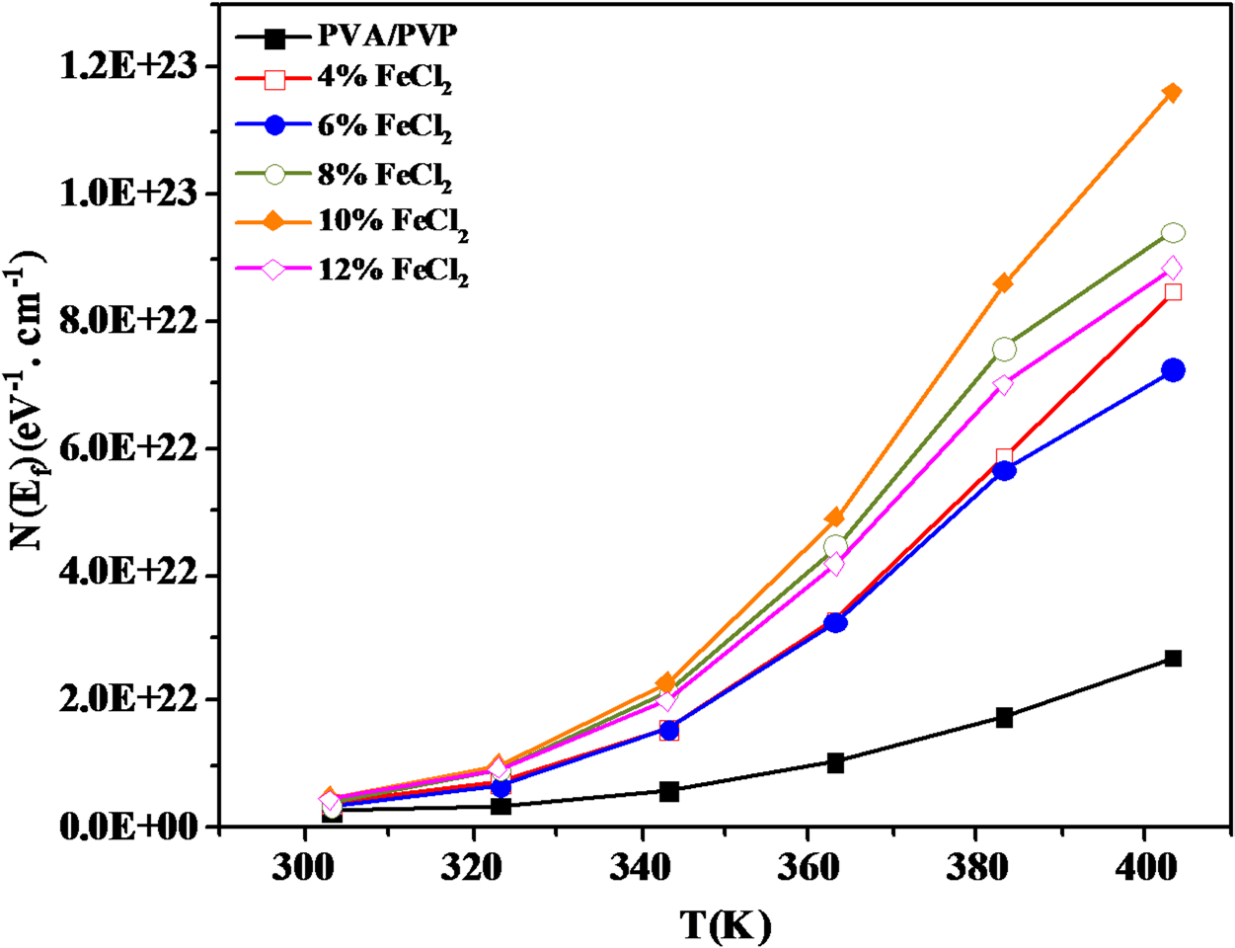
N(E_f_) temperature dependence for PVA/PVP blend filled with different concentrations of hydrated FeCl_2_ at a selected frequency (100 kHz).

N(E_F_) is the density of states at the Fermi level, v_ph_ is the phonon frequency, and α is the exponential decay parameter of the wave functions of the localized states. We can obtain the value of *N*(*E*_*F*_) by putting the values of α^−1^ = 10 Ǻ and *ν*_*ph*_ = 10^12^ s^−1^^54,74^ in Eq. (9). Figure 17 illustrates the variation in density of states with temperature for PVA/PVP/FeCl_2_ composite samples. The graph indicates that the N(E_F_) values increase as the temperature rises for all FeCl_2_ doping levels in the polyblend. This implies that as temperature and doping levels increase, the number of carriers participating in the conduction also increases.

### Nyquist plots

**Fig. 18:**
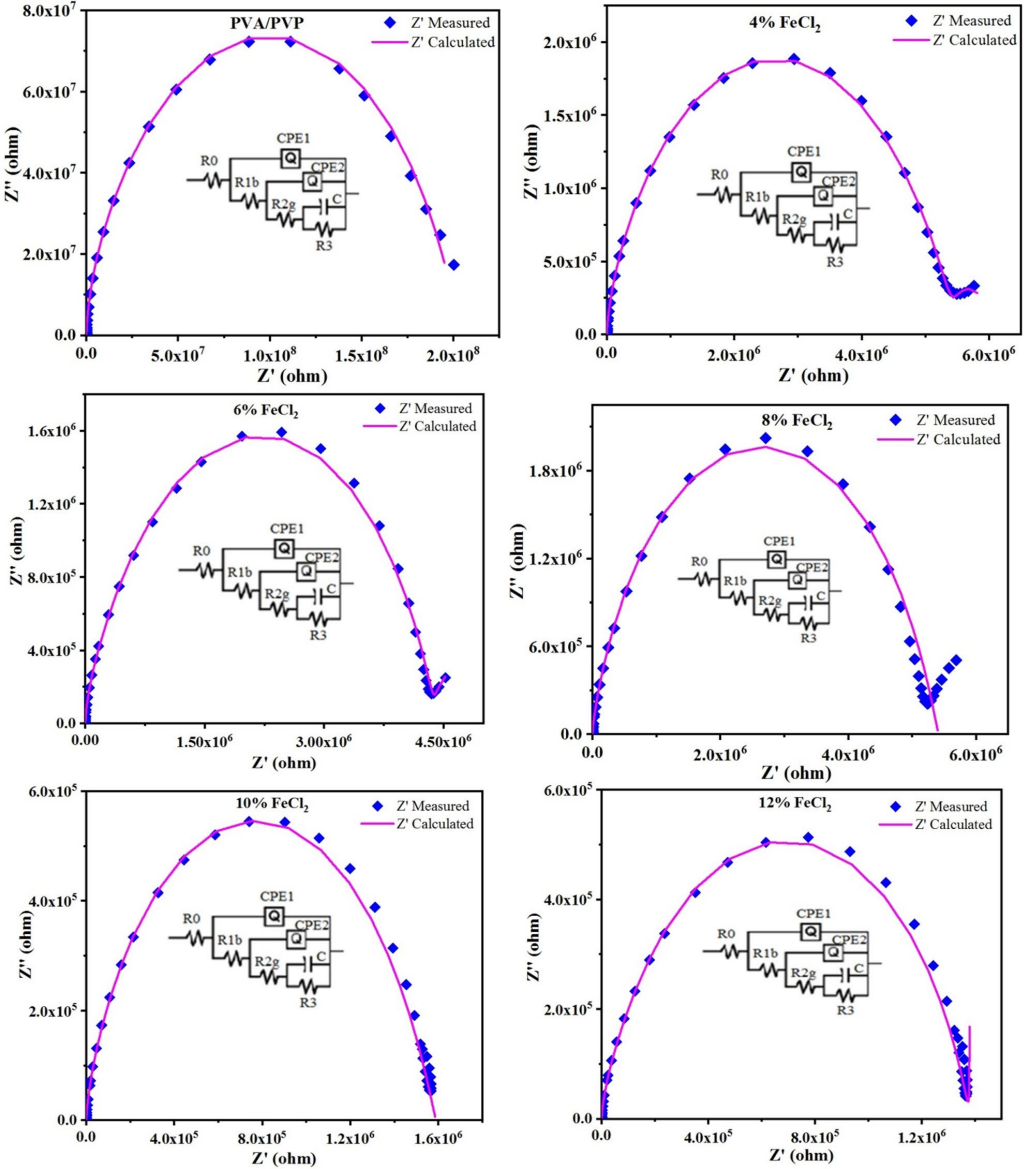
Equivalent circuit fitting by Z simpWin software for PVA/PVP and PVA/PVP/FeCl_2_ composites.

A material’s impedance comprises real (Z) and imaginary (Z) components, which can be visualized on a Nyquist plot. This plot depicts the impact of grains, grain boundaries, electrode material contact, and conduction processes. The Nyquist graphs for the PVA/PVP blend and PVA/PVP/FeCl_2_ composites for frequencies between 100 Hz and 2 MHz are shown in Fig. 18. Only one semicircle was observed in the PVA/PVP blends and PVA/PVP/FeCl_2_ composites. This suggests that there is only one relaxation time constant for the polarization of these materials^75^. The arc radius of the semicircle indicates the composites’ resistance. The capacitance value can be found using the formula ωτ = ωRC = 1, where ω is the peak angular frequency and τ is the relaxation time for a parallel RC circuit^76^. The constant phase element (CPE), denoted by Q, is shown by the depressed semicircles, which depict the non-Debye capacitance structure^77^. The following relationship describes the impedance of the CPE.

11 $${Z}_{CPE}= \frac{1}{Q {(i\omega )}^{n}}$$

At ω = 1 rad/s, Q represents the numerical value of 1/|Z|, while n indicates the phase of the elements and signifies the degree of deviation from a pure capacitor. The Constant Phase Element (CPE) mimics a pure resistor when n = 0 and a pure capacitor when n = 1. To evaluate the values ​​of R, Q, and n for PVA/PVP blends and all PVA/PVP/FeCl_2_ composites at room temperature, Nyquist plots are generated using ZsimpWin software. Non-Debye-type behavior is revealed by the plots’ semicircle arc. The equivalent circuit consists of a resistance and capacitors in parallel combination, along with a CPE. Due to the random positioning of the doped FeCl_2_ in the PVA/PVP chain, which distorts the mixed matrix, these components are present in the circuit. Table 5 displays the resistive and capacitive components obtained from the Z simpWin software for PVA/PVP blend and PVA/PVP/FeCl_2_ composites. The impedance behavior of the composites was simulated using an electrical equivalent circuit model, R(Q(R(Q(R(CR))))), to fit the experimentally collected impedance data. The analogous circuit for the current investigation is the parallel combination of bulk capacitance Q_1b_, CPE_1_, and bulk resistance R_1b_, along with a series resistor R_0_, which represents the bulk resistance of the polymer and the electrolyte. The impedance plot displays two semicircular arcs, the low-frequency arc exhibiting the grain boundary effect and the high-frequency arc corresponding to the bulk property. Because of charge transfer at low frequencies, the equivalent circuit is thus provided by the series of parallel combinations of *R*_*1b*_*Q*_*1b*_ (R_1b_CPE_1_) and *R*_*2g*_*Q*_*2g*_ (R_2g_CPE_2_), where *R*_*2g*_ and *Q*_*2g*_ are the grain boundary resistance and capacitance in parallel connection with resistor R_3_ and capacitor C. With varying values for C, R, and Q, the analogous circuit has the same impedance behavior as a pure capacitor.

**Table 5:** Resistive and capacitive components of grains and grain boundaries for PVA/PVP and PVA/PVP/FeCl_2_ composites equivalent circuit R(Q(R(Q(R(CR))))).

Circuit components	0% FeCl_2_	4% FeCl_2_	6% FeCl_2_	8% FeCl_2_	10% FeCl_2_	12% FeCl_2_
R_0_ (ohm)	0.02608	0.0004809	0.1267	0.6241	2.128	2.897
CPE_1_ (S.sec^0.8)	3.521E−18	5.002E−10	7.672E−10	4.417E−10	4.185E−7	7.991E−10
R_1b_ (ohm)	11.8	28.96	4.153E6	5.165E6	5.275E28	1.334E6
CPE_2_ (S.sec^0.8)	5.777E−9	3.059E−7	5.206E−5	9.653E−5	0.008742	1.385E−5
R_2g_ (ohm)	2.528E−5	5.193E6	9.442E9	1.856E21	2.754E15	7.536E12
Capacitance C (F)	1.74E−10	3.205E−6	9.514E−7	0.0002515	1.476E−20	4.088E−6
R_3_ (ohm)	9.745E9	1.364E9	162.5	0.01429	1.926E12	0.000176

### Antibacterial activity

**Fig. 19:**
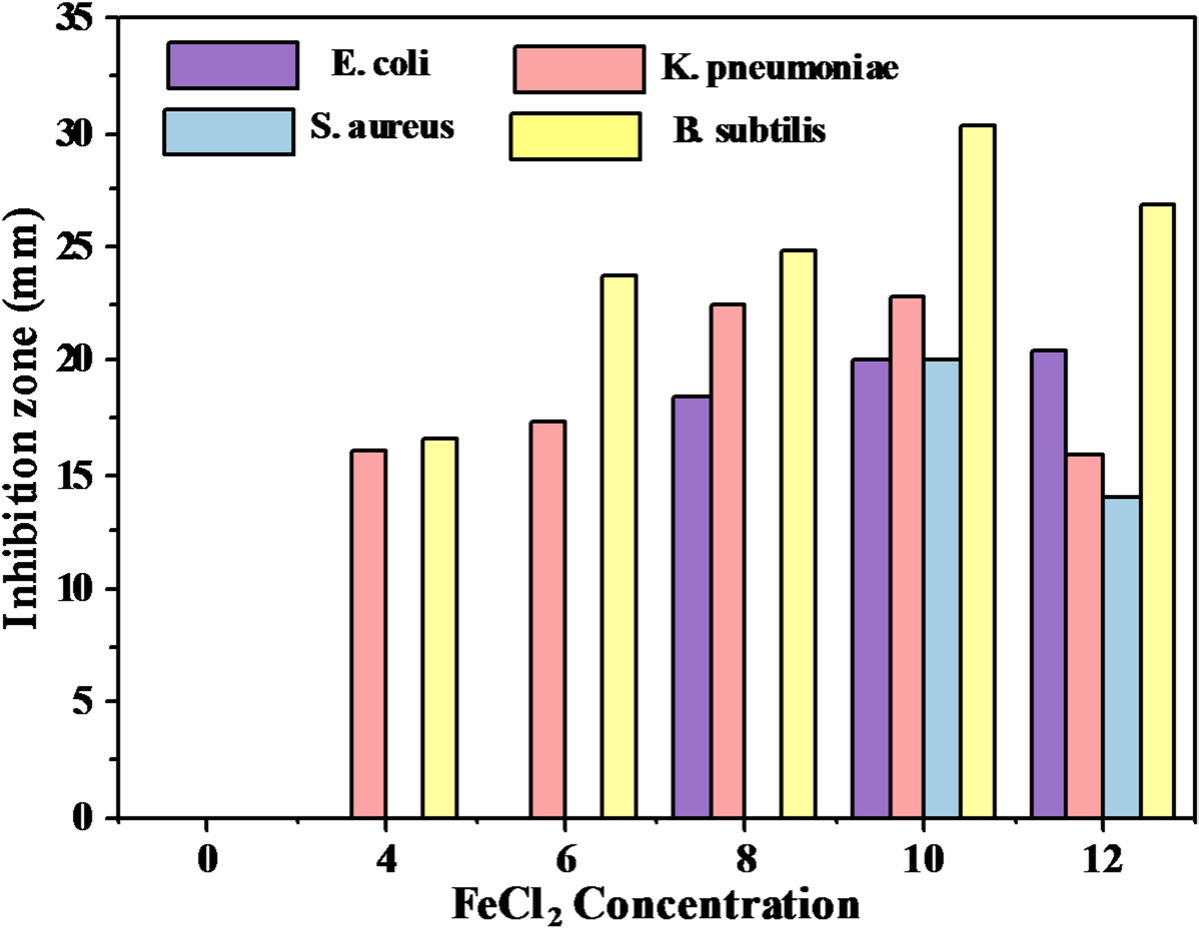
Inhibition zone for PVA/PVP and PVA/PVP/FeCl_2_ composites.

Pure PVA/PVP and PVA/PVP/FeCl_2_ composite films were evaluated for their antibacterial activity against gram-positive (S. aureus and B. subtilis) and gram-negative (E. coli and K. pneumoniae) pathogens using nutritional agar medium. The inhibition zone diameter (IZD) for all investigated samples was evaluated on a millimeter scale in three separate experiments. Following plate incubation, the inhibition zone (mm) of the films was also visible in Table 6. The pristine PVA/PVP blend sample does not exhibit germicidal properties against either gram-negative or gram-positive microorganisms. In contrast, PVA/PVP/FeCl_2_ composite films exhibit a positive response, showing germicidal properties against both Gram-negative and Gram-positive organisms (Fig. 19). This study demonstrated that a concentration of 10 wt% FeCl_2_·4H_2_O yielded the maximum IZD value. All composite films display an inhibition zone for K. pneumoniae and B. subtilis bacteria.

**Table 6:** Inhibition zone for FeCl_2_ against the tested bacterial strains.

FeCl_2_ Concentrations	Zone of inhibition (mm)			
	E. coli	K. pneumoniae	S. aureus	B. subtilis
0	NA	NA	NA	NA
4	NA	16.0 ± 1.0	NA	16.6 ± 0.6
6	NA	17. 3 ± 0.6	NA	23.8 ± 0.6
8	18.5 ± 0.6	22.4 ± 0.6	NA	24.9 ± 0.6
10	20.0 ± 1.0	22.8 ± 0.6	20.0 ± 1.0	30.3 ± 0.6
12	20.5 ± 0.6	15.9 ± 0.6	14. 1 ± 0.6	26.8 ± 0.6

In contrast, there was no apparent inhibiting zone against S. aureus and E. coli at concentrations of 4, 6, and 8 weight percent FeCl_2_·4H_2_O. The effectiveness of FeCl_2_ as an antibacterial agent was assessed, demonstrating that, for all pathogenic bacteria except 12% of the FeCl_2_-treated samples, the zone of inhibition increased in proportion to the FeCl_2_ concentration. The antibacterial activity of FeCl_2_ is concentration-dependent for all organisms. However, it was more potent at higher doses. The gram-positive and gram-negative bacteria under examination exhibit varying sensitivity to FeCl_2_. The primary reason for this is the thickness of the peptidoglycan layer and its response, which might hinder the movement of FeCl_2_ through the bacterial cell wall^78,79^. The suggested mechanism for the antibacterial activity of these polymeric composites corresponds to the generation of reactive oxygen species (ROS) and the release of ferrous ions (Fe^2+^) from FeCl_2_. These composites have exceptional antibacterial properties, which make them very appealing for use in medical packaging applications where product security and hygiene are crucial to prevent damage to bacterial cells, causing them to disintegrate^80,81^ eventually. Because of their exceptional antibacterial properties, PVA/PVP/FeCl_2_ composites are very appealing for use in medical packaging applications where product security and cleanliness are crucial.

## Conclusions

Using the solution casting method, PVA and PVP were combined in a 1:1 ratio to create polyblend films. FeCl_2_ was added to these samples in various quantities. The PVA/PVP blend’s degree of crystallinity was reduced by the addition of FeCl_2_, according to the XRD study. This suggested that the metal salt significantly impacted the polymer blend’s characteristics. The FT-IR spectral analysis revealed significant changes in band position and intensity compared to the pure blend, providing strong evidence of an interaction between the polymer blend and the metal salt. The samples’ thermal stability was improved by adding FeCl_2_ to the PVA/PVP matrix, suggesting that the material might withstand greater temperatures without degradation. The composites’ energy band gap ranged from 3.07 to 2.31 eV, according to the UV–Vis results, which demonstrated their good optical properties.

Because of this, the material can be used in a variety of optoelectronic applications. The composite samples exhibited paramagnetic behavior, increasing saturation magnetization as the FeCl_2_ content increased. This suggests that the material could be used in magnetic applications. The AC electrical conductivity data showed that the system’s conductivity increased with temperature and the addition of metal salt. The examined system utilizes a hopping mechanism in electrical transport, as indicated by the activation energy values ​​that are reflected in the conductivity and dielectric modulus. This information provides insight into the material’s underlying electrical characteristics. The mean value of the binding energy, W_M_, decreases with increasing temperature and FeCl_2_ content. The equivalent electrical circuit of the composite films was determined by examining the impedance components Z′ and Z″. The current system conforms to non-Debye relaxation. Composite films demonstrated an adverse effect against gram-positive bacteria (S. aureus and B. subtilis) as well as gram-negative bacteria (E. coli and K. pneumoniae) when the antibacterial activity was examined. This implies that the substance is a good fit for application in medicine.

## Data Availability

The datasets used and/or analyzed during the current study are available from the corresponding author upon reasonable request.
